# From “Airway scares me” to “I would say I’m pretty comfortable”: quality improvement for reducing time to obtain equipment for adult advanced airway management in a rural emergency department

**DOI:** 10.1007/s43678-024-00828-8

**Published:** 2025-01-04

**Authors:** Ava Butler, Michael Chen, Shruti Kaushik, Terra Lee, Liam Raudaschl, Audrey Giles

**Affiliations:** 1 Faculty of Emergency Medicine, University of British Columbia, Duncan, BC Canada; 2 Medical Biochemistry (Island Health), UBC, University of Victoria, Victoria, BC Canada; 3 Island Health, Victoria, BC Canada; 4 Cowichan District Hospital, Duncan, BC Canada; 5 University of Victoria, Island Medical Program, Victoria, BC Canada; 6 School of Human Kinetics, University of Ottawa, Ottawa, ON Canada

**Keywords:** Rural emergency medicine, Airway management, Translational simulation, Quality improvement, Médecine d’urgence rurale, Gestion des voies respiratoires, Simulation translationnelle, Amélioration de la qualité

## Abstract

**Background:**

Management of the adult airway is one of the most stressful and time-critical procedures in emergency medicine. In the Cowichan District Hospital, a rural hospital in British Columbia, Emergency Department (ED) staff were uncomfortable with acquiring the equipment needed for adult advanced airway management and the mean length of time to acquire the equipment was 319 s. The aim of this quality improvement (QI) project was to decrease the time to obtain the equipment needed for adult advanced airway management by nurses and physicians in the Cowichan District Hospital ED to less than 90 s by May 2023.

**Methods:**

The Institute for Healthcare Improvement model of improvement was used to reduce the amount of time required to obtain the equipment for adult difficult airway management in the ED, which was measured using a standardised tabletop simulation every 2 weeks. Change ideas included using a colour-coded airway cart and employing translational simulation. Qualitative interviews with emergency department staff after intubations of patients in the ED captured process measures by examining provider comfort.

**Results:**

From December 2022 to May 2023, the mean time to obtain equipment for adult advanced airway management decreased from an initial value of 319 s to 76 s, a 76% improvement from the baseline. Qualitative interviews obtained pre-intervention, mid-intervention and post-intervention reflected themes of initial discomfort, shifting discomfort to comfort and finally to comfort.

**Conclusion:**

The change ideas of using a colour-coded airway cart and translational simulation were associated with a reduction in time to obtain equipment for management of the adult advanced airway as well as improved provider comfort with the procedure in a rural ED.

## Résumé

**Contexte:**

La gestion des voies respiratoires adultes est l’une des procédures les plus stressantes et les plus chronophages en médecine d’urgence. À l’hôpital de district de Cowichan, un hôpital rural en Colombie-Britannique, le personnel du service des urgences (DE) n’était pas à l’aise avec l’acquisition de l’équipement nécessaire pour la gestion avancée des voies respiratoires des adultes et la durée moyenne d’acquisition de l’équipement était de 319 secondes. Le but de ce projet d’amélioration de la qualité (QI) était de réduire à moins de 90 secondes, d’ici mai 2023, le temps nécessaire pour obtenir l’équipement nécessaire aux infirmières et aux médecins pour la gestion avancée des voies respiratoires des adultes au DE de l’hôpital de district de Cowichan.

**Méthodes:**

Le modèle d’amélioration de l’Institute for Healthcare Improvement a été utilisé pour réduire le temps nécessaire à l’obtention de l’équipement nécessaire pour la gestion des voies respiratoires difficiles chez les adultes dans l’ED, qui a été mesuré au moyen d’une simulation normalisée toutes les deux semaines. Les idées de changement comprenaient l’utilisation d’un chariot de voies respiratoires codé par couleur et l’emploi de la simulation translationnelle. Entrevues qualitatives avec le personnel du service d’urgence après l’intubation des patients dans la DE, qui ont capturé les mesures de processus en examinant le confort du fournisseur.

**Résultats:**

De décembre 2022 à mai 2023, le temps moyen d’obtention d’équipement pour la gestion avancée des voies respiratoires adultes a diminué, passant d’une valeur initiale de 319 secondes à 76 secondes, soit une amélioration de 76 % par rapport à la référence. Les entretiens qualitatifs obtenus avant, au milieu et après l’intervention ont reflété des thèmes de malaise initial, passant du malaise au confort et enfin au confort.

**Conclusion:**

Les idées de changement consistant à utiliser un chariot de voie aérienne et une simulation translationnelle codées par couleur étaient associées à une réduction du temps nécessaire pour obtenir l’équipement nécessaire à la gestion des voies aériennes avancées chez l’adulte ainsi qu’à une amélioration du confort des fournisseurs avec la procédure dans un DE rural.

## Clinician Capsule

**Table:** 

***What is known about the topic?***	
Rural ED MDs and RNs can take too long and be uncomfortable with obtaining difficult airway equipment.	
***What did this study ask?***	
Decrease the time to obtain equipment needed for adult advanced airway management to less than 90 s by May 2023.	
***What did this study find?***	
This quality improvement project found a 76% decrease in the time to obtain airway equipment and increased provider comfort.	
***Why does this study matter to clinicians?***	
An airway cart and translational simulation could make obtaining difficult airway equipment faster and more comfortable for rural ED providers.	

## Introduction

### Problem description

In emergency medicine, adult advanced airway management can be required with little warning and difficulty in obtaining definitive airway control cannot always be predicted [1]. Longer time to definitively manage the difficult airway means higher risk of adverse events for patients [2]. In the Cowichan District Hospital ED, the team for this procedure includes nurses, physicians and RTs. Given there is only 1 respiratory therapist on site responsible for covering the ED, ICU and inpatients, gaps in respiratory therapist availability to the ED exist in the case of short staffing, staff illness or if the respiratory therapist is occupied with another patient. When a respiratory therapist is not available, MDs and RNs sometimes struggled to find the equipment needed for advanced airway management, increasing the stress of the procedure and potentially delaying airway management.

### Available knowledge

The use of a cart for organisation of airway equipment is a practice that has been described in multiple settings [3–5]. Crimes et al. [4] argue standardised airway trolleys must be rapidly available in any area of the healthcare environment where advanced airway management could be required. The use of colour-coded drawers for a difficult airway cart has been described as a “cognitive aid…(that) can easily and powerfully enhance the management of an unanticipated difficult airway” [5].

Translational simulation was used as a change idea in this QI project. Translational simulation uses representation of clinical scenarios to “directly improve health care processes and outcomes” [6]. The purpose of translational simulation sets it apart from traditional simulation; instead of changing individual or team performance, it is used to identify areas of the system that need improvement and provides simulation-based interventions to improve patient care. Translational simulation has been suggested as a tool for QI [7, 8]. Nickson et al. (2021) offer translational simulation as an effective tool for examining medical environments, with a focus on “equipment, medication, (and) physical space usage” [9].

### Rationale

The arrangement of the physical workspace and the formation of a mental model are two key areas identified as potential latent safety threats in resuscitation [10]. Optimising equipment and teamwork for adult airway management in a rural ED allows physicians and nurses to more efficiently adapt for unexpected difficulty in the absence of a respiratory therapist. This was predicted to improve provider comfort with obtaining equipment and the performance of advanced airway management. A patient partner of the project noted how improved organisation of equipment improves the health care experience for the patient, noting, “For anyone who is even slightly coherent to have people run around to get the equipment to help them to breathe is unbelievable…it should not be happening.”

### Specific aims

The aim of the QI project was to decrease the time to obtain the equipment needed for adult advanced airway management by nurses and physicians in the Cowichan District Hospital ED to less than 90 s by May 2023.

## Methods

### Context

The Cowichan Valley is included as a rural area “C” under the Rural Retention Program from the Government of British Columbia [11]. The population in the 2021 census was 89,013[12]. Cowichan District Hospital ED census is approximately 33,000 visits per year [13]. There are approximately 20 permanent physicians who work in the ED, 6 RTs and approximately 50 nurses. The hospital has in-house ED physician coverage ranging from 1 to 4 physicians depending on the time of day. The number of intubations in the ED per year is not measured by the hospital.

### Interventions

Using the Institute for Healthcare Improvement Model for Improvement [14], Plan-Do-Study-Act (PDSA) cycles were used to evaluate the impact of change ideas on project measures (Table 1). Change ideas were gathered from suggestions from ED staff using the liberating structures 25/10 crowdsourcing technique [15] and selected based on feasibility and predicted impact in comparison with literature review. The change ideas that were the focus of the QI project were creating a portable colour-coded airway cart and translational simulation.

**Table 1:** Plan-do-study-act cycles to decrease time to obtain advanced airway

PDSA cycle	Plan	Do	Study	Act	Time
Airway cart	Improve equipment access for staff	Colour-coded airway cart created and placed in trauma room	Initial decrease in time to obtain equipment	Adapt: nurses request cricothyroidotomy equipment bundled and drawer to be re-labelled	Jan. 16, 2023
Translational simulation 1 and 2	Translational simulation will improve the team’s ability to manage difficult airway	Translational simulation case: advanced airway management in pneumonia patient with a head injury	Mean time to obtain equipment decreases to 85 s	Adapt: cases for simulation adjusted with increased complexity to encourage use of all the cart drawers	Jan. 24, 2023 Feb. 9, 2023
Translational simulation 3 and 4	New translational simulation case will encourage cart use	Translational simulation: advanced airway management in a patient with anaphylaxis	Mean time to obtain equipment decreases to 63 s	Adopt: ongoing interdisciplinary simulation with advanced airway management as component	Mar. 6, 2023 Apr. 3, 2023

#### Airway cart

An unused cart was obtained from hospital stores. The drawers were colour-coded with duct tape. Process mapping for advanced airway management in the Cowichan District Hospital ED was consolidated into a flowsheet and reviewed by the nurse educator, emergency and anaesthesia physicians. Drawers were stocked by colour to correspond with the airway flowsheet and were adjusted through the PDSA cycles. For example, in initial testing, there was confusion about which equipment was needed for cricothyroidotomy, so the equipment was bundled and labelled so it could be obtained with one hand.

#### Translational simulation

A total of 4 translational simulation sessions were undertaken. These were interdisciplinary simulations of emergency medicine cases requiring airway intervention. They were held monthly between January and April, 2023. Attendance at simulation was compensated sessionally for physicians and as education time for nurses. Each 2-h simulation was attended on average by 5 nurses and 4 physicians.

### Measures

**Fig. 1:**
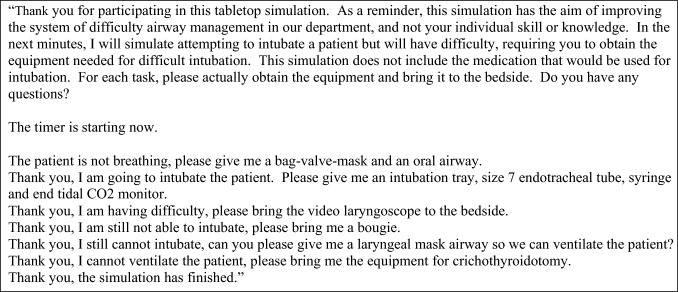
Prompts for tabletop simulation

Time required to obtain the equipment for adult difficult airway management in the ED measured using a tabletop simulation with standardised script (Fig. 1) was the outcome measure. Provider level of comfort in obtaining equipment and airway management process was the process measure. The percentage of staff that attended non-simulation educational events was also tracked as a balancing measure.

Baseline measurements of time to obtain equipment were taken, and then taken again approximately every 2 weeks during the project timeline. RNs and MDs were selected for these measurements in the following manner: the MD closest to the end of their shift, one RN from the “streaming” and one from the “cardiac” positions for that shift were invited to participate. Measurements were taken with staff independently, not in groups. An average of 4 staff were tested at each measurement interval. 24% of the measurements were during dayshifts, 16% during evening shifts and 60% during nightshifts. The same author (AB) administered the script to ensure standardisation of timing of the prompts.

**Fig. 2:**
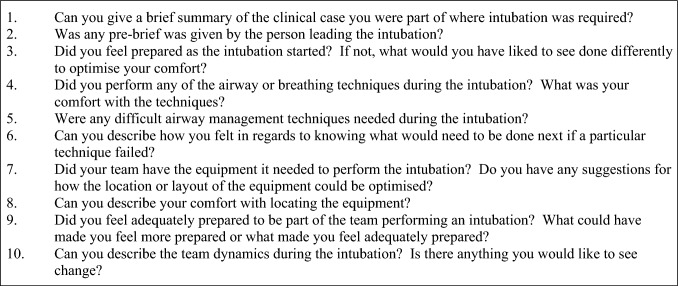
Semi-structured interview guide

Qualitative semi-structured interviews with doctors and nurses were conducted monthly during the project to track provider comfort with actual cases of intubation in the ED (Fig. 2). There were 7 participants (4 doctors, 3 nurses) at 3 time points: 1 month before the intervention (3 participants), 1 month into the intervention (2 participants), and 1 month after the intervention (2 participants). The interviews were recorded and transcribed verbatim.

### Analysis

Quantitative data for time to obtain equipment for advanced airway management for participating providers (n = 29) were analysed from December 2022 to April 2023. Data analysis was conducted with run and Shewhart charts constructed in MS Excel and SQC pack, which were used to study change in time to obtain equipment. These charts were analysed using standard rules to detect signals of non-random variation.

Braun and Clarke’s 6-step framework for thematic analysis [16] was used to inductively analyse the qualitative interviews to understand changes staff members’ comfort as the project progressed. The lead author familiarised herself with the transcripts. She then manually coded the transcripts. She then grouped the codes to develop themes and then reviewed them to ensure them reflected the overall data. Subsequently, she named the themes. The codes “unprepared,” “unfamiliar,” “chaos,” and “intimidating” resulted in the theme “discomfort.” The codes that produced a theme of “comfort” were “confidence,” “experienced,” “organized,” “optimized,” “prepared,” and “easy.”

### Ethical considerations

The Island Health QI Ethics Decision Making Tool was used to screen the project for ethical concerns. It was reviewed by the Island Health ethics team, determined to be a low-risk QI project and thus exempt from research ethics board review.

## Results

### Quantitative results

Baseline data collected prior to the start of this QI initiative were obtained from 4 ED nurses and physicians, who took a mean of 319 s to obtain the equipment needed to manage the adult airway.

**Fig. 3:**
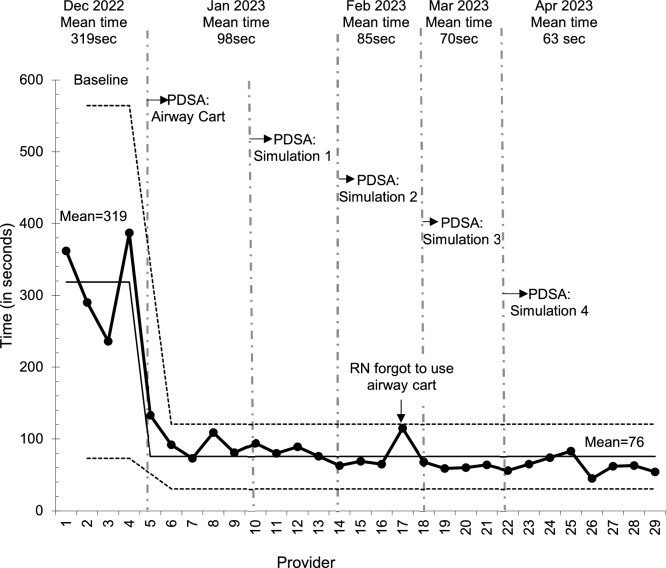
Shewhart (I-chart) showing time taken by providers to obtain equipment for difficult airway management during airway tabletop simulation (Cowichan District Hospital—Emergency Department, December 2022–April 2023)

An I-chart was constructed to study variation in time to obtain equipment for individual providers (Fig. 3). Success in reducing time became evident following implementation of the airway cart and first translational simulation, with mean time taken to obtain equipment decreasing by 76% from baseline to a mean of 76 s.

The percentage of staff attending non-airway-related education events did not decrease during the time of the project averaging 18% of staff attending education from September to December 2022 and 36% from January to April 2023. As this was a balancing measure to ensure no adverse decrease in attendance at alternative educational events, no statistical tests were applied to this measurement.

### Qualitative results

Qualitative interview data over the course of the project demonstrated a shift in codes and thus themes from discomfort to increasing comfort and finally comfort with both airway equipment and airway process.

### Pre-intervention: discomfort

Pre-intervention, the nurses and physicians expressed their discomfort with both the equipment and the process of managing a more complex adult airway. In pre-intervention team consultations, Nurse D stated, “Airway scares me…I always feel disorganized.” Nurse B reflected in a semi-structured interview pre-intervention, “I was asked to find the equipment for intubation because, obviously, the doctor was running the show, and I had no idea what to even look for. So, I passed it on to a colleague, who also didn’t really know what they were looking for.” Physician A noted about an intubation prior to the intervention, “I think everybody that day was unfamiliar with the intubation equipment” and Nurse C commented, “I want to be a bit more aware of what the next step is going to be for me at the head of the bed.”

### Mid-intervention: shift from discomfort to comfort

As the airway cart was introduced and the translational simulations were started, there was a shift in themes from discomfort to comfort with the airway equipment and process. After a difficult intubation in the ED, Physician C noted during the mid-point of the intervention, “The airway cart was super fantastic, I would 100% want to have that again.” This physician also noted an improvement in the process of intubation, saying, “We’ve done a lot of team-building exercises…and simulations, which I think have really optimized the team.” There was still some discomfort noted by Physician B during the mid-point, who said, “It’s…an issue if there is no (respiratory therapist) and (the nurses) have to get everything…making sure that it’s all accessible is the tricky part sometimes.”

### Post-intervention: comfort

Post-intervention, nurses and physicians expressed comfort with the airway cart and process. Nurse D stated, “That airway cart is perfect. Because everything is there and if you forget something or if someone is a bit confused you can say 'in the green one!’ and you can find it.” Physician D stated, “I actually spent some time this week looking through (the airway cart)…and it sure increases my confidence.” When asked about comfort with airway equipment and process post-intervention, Nurse D stated, “I would say I’m pretty comfortable. It’s pretty clear.”

## Discussion

### Interpretation of findings

Colour-coded airway carts and translational simulation were effective changes in this rural emergency department in decreasing the amount of time needed for emergency medicine providers to obtain the equipment needed for adult advanced airway management. These practises, translated from urban centres, were implemented using an interdisciplinary, team-based and context-specific approach. This led not only to the quantitative improvement in time to obtain equipment but also to the qualitative process data demonstrating an improvement in provider comfort. Increased efficiency in obtaining equipment and increasing provider comfort are predicted to improve patient and healthcare provider experience and safety during advanced airway management in this rural emergency department.

### Comparison to previous studies

The aim of both translational simulation and QI is to improve patient care [8]. Translational simulation has up to this point been described in urban and tertiary care centres [16–20]. The literature did not contain any examples of translational simulation in community or rural hospitals. In hospitals all along the rural spectrum, there are decreased resources compared to major metropolitan hospitals for research [21], simulation [22], and QI [23]. In this project, local expertise and team infrastructure were used to create a successful, community-based QI initiative using the relatively new model of translational simulation.

### Strengths and limitations

This project was developed in a rural ED with limited research and QI resources. The airway cart was created from existing materials and did not require additional materials. Translational simulation in our hospital was integrated into our existing simulation program at no added cost and did not adversely affect attendance at other educational events.

The quantitative measurements in this project were conducted in a well-controlled simulated environment, which is a surrogate for real patient outcomes—not an actual patient outcome itself, thus directly improved patient care is not demonstrated in this project. There were only 4 measurements at the baseline, which is smaller than the recommended number of data points. The authors chose to proceed with the intervention despite the low number of baseline measurements because there was a clear demonstration of a problem, there was contextual knowledge to support the baseline that was measured and the alternative would have been to delay the intervention, which may have been detrimental to patient care.

### Clinical implications

Longer time to definitively manage the difficult airway means higher risk of adverse events for patients [2]. Decreased time to obtain the equipment to manage a difficult airway and increased health care provider comfort are expected to improve the management of adult airway emergencies in the Cowichan District Hospital ED. Ongoing orientation of new staff to the airway cart and integration of difficult airway cases into our department’s monthly interdisciplinary simulation will be used to sustain the impact of this project.

### Research implications

The creation of colour-coded airway carts in rural emergency departments has the potential for low cost and relatively low barrier spread to similar sized hospitals. Translation simulation is an emerging tool in QI [8]. Ongoing QI work in rural hospitals can help to identify other areas in which translational simulation can be applied. Increasing knowledge exchange between similarly sized and resourced Canadian hospitals may help to decrease duplication in QI in this context and improve spread.

## Conclusion

Rural emergency teams must be ready at all times to perform the time-critical procedure of adult advanced airway management. Difficulty in performing the procedure cannot be predicted, so teams must be adept at obtaining equipment and working together to obtain advanced airway access. In this quality improvement project, use of a colour-coded airway cart and translational simulation as change ideas in a rural ED was associated with a reduction in the amount of time needed for emergency medicine providers to obtain the equipment needed for adult advanced airway management by 76% to a mean to 76 s and increased the comfort of RNs and MDs without requiring additional equipment or infrastructure. This project could be undertaken in similarly sized EDs.

## Data Availability

The data from this quality improvement project are available from the corresponding author, AB, upon reasonable request.
